# The Spoken Word and the Printed Page

**Published:** 1917-09-08

**Authors:** 


### THE TEACHING VALUE OF LECTURES.

### The Spoken Word and the Printed Page.

In the medical curriculum teaching by means of lectures occupies a very prominent position. Each subject included in the educational scheme has its prescribed number, and these the official teacher must duly deliver. The student in his turn must attend a certain proportion of each series, and must show formal proof of such attendance before he can be admitted to the corresponding examination. Practical instruction in laboratories and in hospital has nowadays a large and growing place in the curriculum, but the lecture continues to hold its own. It is heard at the outset, and ceases only when the student has completed the full measure of the prescribed classes. The method has its critics, and these do not invariably avoid the language of scorn. It may be that the critics are an increasing body, and that they can find larger opportunities for their destructive ambitions. So far, however, they can boast but little practical success, and the student still feels a power which seats him on the pupil's bench and bids him lend to the official prelection at least the appearance of an attentive ear. Whatever be the value of the individual performance none may escape either the summons or the hour.

Like many other institutions which show an obstinate front to repeated assaults, the lecture can boast a long and respectable history. Hence it comes with a presumption in its favour, and there would seem reason for admitting that, though possibly it may not be justified or necessary to-day, it has served a worthy purpose in the past. Such a tribute is allowed even by its detractors, who, however, are none the less energetic in denouncing the modern survival. Of course, there was a time when the spoken word was necessary—when, indeed, it was almost the only possible word. Before the days of printed books the student had perforce to sit at the feet of the master and to listen to the words of wisdom which fell from the professorial lips. The lecture was the one available means of teaching, and both teacher and taught had to make the best of it. But nowadays, and indeed for many a long year, no such necessity exists. Books abound, and some are good and many cheap. All the information which a teacher has to convey may be presented on the printed page with tricks of type and paragraph apt for emphasis and for orderly perspective. Diagrams and illustrations add further values, and, so it is argued, it is the book and not the lecture which should be the modern educational instrument. The latter survives by force of precedent and of tradition, but it is unnecessary in the presence of books, and, as a matter of fact, leads to much waste of time and strain of temper. The student would be better occupied in his study and the professor in his library or workshop. Such is the indictment urged against the lecture as a teaching method. It has exhausted its usefulness, and may now profitably be placed upon the shelf, being as much out of date in the world of education as is the knight in armour on the modern battlefield. Both to the one and the other let us pay due respect and then turn without delay to the more efficient apparatus which progress has placed at our disposal.

Now it may at once be admitted that if the sole or even the main purpose of the lecture is to convey information little can be said for the persistence of the lecturing system. This purpose, let it be freely allowed, is much more successfully attained by printed books. Still more, candour compels an acknowledgment of the existence of lecturers who appear to forget that, in an age of cheap printing, some achievement other than the mere recital of facts is necessary to justify their existence. They continue the ancient tradition and thus may not escape the reproach which this nowadays

**THE TEACHING VALUE OF LECTURES**—(*continued*).

incurs. It is, indeed, a ridiculous and pathetic picture to witness in the twentieth century a class-room filled with students each of whom is scribbling in his notebook as best he may the sentences read off from a manuscript by a more or less archaic professor. The performance is as out-of-date as soothsaying or witchcraft. Most certainly the principal performer is not needed, for his place could be effectively taken by a gramophone. To the student the lecture on this plan is a mere weariness to the flesh, and little wonder is it that he shirks the discipline as often as this is possible, or, failing flight, is driven to pitch-and-toss or to slumber in the shelter of the back benches. Nor for the industrious and attentive does the torment terminate with the official hour. Loaded with a hastily and badly written manuscript containing almost necessarily a proportion of errors, he seeks in his study to arrange this in some degree of form and order. There is no clear arrangement of paragraphs, no grades of type, no emphasis of light and shade, no pictorial helps. At the best all he has for his pains is a dictated lesson entirely free from the mechanical aids and ordered system of the ordinary text-book. In face of lectures of this order it is little wonder that lecturing as a method of teaching falls into derision, and is condemned as a useless, absurd, and incongruous anachronism. The printed book easily carries the day, and the bored and wearied student flies the class-room except in so far as compulsion insists on his presence. The demand is for so many attendances, and these earn the official voucher. But as for any other advantages none may be recorded. The experience is wholly wearisome, depressing, and soul-destroying, and its victims feel it to be a mere burden of useless and official tyranny. For these uncorrected exercises in dictation which are unjustly dignified with the name of lectures none but their authors—and not always these—can find any valid defence or excuse. They have had their ancient day; let them summarily cease to be.

To make these admissions, however, is not to accept the contention that the lecturing system is useless and unnecessary, and ought to be swept away. This claim might be justified were the object of education solely the implantation of facts in the student's brain and memory, for here the text-book satisfies the full ambition, and it does this infinitely better than a manuscript of lectures. But teaching or education has no such limited aim. Its purpose is not merely to inform the mind but also to develop and strengthen it; to cultivate habits of thought, reasoning, and mental order; and to encourage a capacity for judgment, for the weighing of evidence, and for a broad and balanced outlook. It may be said that these ends can be secured, and have in actual cases been secured, through the agency of books; and Carlyle, who in vigorous language depicted himself as having suffered many things at the hands of lecturers, proclaimed the "university of the future" to be the library. He was not tolerant of speech, not at least in others, and thus, as a modern critic has put it, "He preached the doctrine of silence in thirty volumes." But to exalt lectures it is not necessary to depreciate books. Nor is it necessary to deny that individual men and women have succeeded in educating themselves to high levels through the agency of books. An educational scheme, however, must be planned for the many and not for the exceptional few, and it must command all the methods which are likely to secure the desired end in the readiest and most effective manner. In such a scheme books necessarily have their place, and their place is a highly important one. They offer the ordered presentation of the knowledge which is to be the groundwork of the student's equipment. But in the very nature of the case this knowledge is set in a somewhat rigid framework. It is severe, inelastic, unemotional, and is apt to present a somewhat forbidding front. Only the quite exceptional writer is able to confer on it the qualities which excite an appetite for acquisition and provide the glow of enthusiasm and the note of human interest. It is almost in the nature of formal and systematic exposition on the printed page that unsympathetic conditions should attend it. That individuals have triumphed over these difficulties is doubtless true, but their labours in so doing have been heavy; and will the many follow their example? Experience hardly provides an affirmative answer.

It is in the circumstances here set forth that the lecture finds its justification and opportunity. Admit that its function as an agent for communicating facts has been superseded by the text-book; there still, however, remains for it a high and noteworthy influence in the educational scheme. But it must succeed in occupying the position reserved for it, and must not hope for respect and survival if it appears as a mere alternative to its modern and more efficient competitor. In this respect it is dead, and decent burial is its appropriate lot. On the other hand, in its own sphere it stands without a rival and none may take its place. It is not mainly with facts, but with the relations of facts that it is concerned. Its purpose is less to convey knowledge than to provide an atmosphere and to suggest an attitude; to introduce a sense of light and shade; to establish an ordered perspective; and to arouse a habit of inquiry and an enthusiasm both for knowledge and for its application. As a modern teacher has phrased it, the object of the lecture is not to fill the notebook but to thrill the soul, and the test of its success is less the addition of new facts to the student's record than the arrival of a new influence in his mental equipment. So regarded the lecture takes on its true dignity and value, and is seen as a method which an educational enterprise cannot afford to neglect. As a cold and colourless announcement of facts it is a burdensome survival which may well be cast into the lumber-room. But let it live up to its opportunity and it may count on an abiding position in which it will claim both the appreciation of the expert and the gratitude of the student.

Perhaps it may be said that these lofty doctrines may be all very well in what may be called

**THE TEACHING VALUE OF LECTURES**—(*concluded*).

a general educational ambition, but that there is little place for them in a subject so severely practical as medicine. What is wanted here, the argument may run, is an acquaintance with the facts of disease and with the methods by which disease may be successfully treated. It is in the dissecting-room, the laboratory, and the hospital ward that the student must be fitted for his work as a physician and surgeon, and with such an end the glowing enthusiasms of the lecturer have little concern. What the public wants from its doctors is the setting of its broken limbs or the relief of its stomach-aches, not a capacity for the contemplation of truth or a mind trained to habits of reasoned inquiry.

Upon such a comment two remarks may be submitted. First, it is a public interest to attract to the practice of medicine men of high mental calibre and wide sympathies. An education offered for this end cannot hope for success if it includes nothing more than a training along strictly utilitarian lines. Other callings will provide a more inviting atmosphere, and medicine will suffer an intellectual decline. If the profession is to be recruited from men of high type and ability, there must be provided in medical life and training not only the means of livelihood and an opportunity for craftsmanship, but also those subjective influences which contribute to mental growth and development, and which order and train intellectual competence. The practical art is essential, but not less so is the mental atmosphere, and it is here that the competent lecturer may grasp his individual chance in the scheme of training. In the second place, medical practice is by no means the mere repetition of experiences to which the student has been introduced during his period of training. However long and thorough this may be, there will await him in practice new situations and fresh problems. What he has to deal with is not diseases and injuries in the abstract, but disturbances in individual men and women, each of whom has his personal peculiarities and idiosyncrasies. He will meet these situations not by recalling events of exactly the same order and degree, but by applying to them good judgment founded upon technical knowledge and sound reasoning. Consequently both the one and the other of these qualities must be provided in his training. He must be, not the slave of rigid rules, but a master of mental methods. It would be, therefore, a cardinal error to exclude from his education agencies which are vital to such an attainment. In a word, the discipline of the lecture-room and the inspiration of the living teacher are not less necessary than the practice of the hospital and the frigid severity of the printed page.

